# Development of a Target Enrichment Probe Set for Conifer (REMcon)

**DOI:** 10.3390/biology13060361

**Published:** 2024-05-22

**Authors:** Raees Khan, Ed Biffin, Kor-jent van Dijk, Robert S. Hill, Jie Liu, Michelle Waycott

**Affiliations:** 1 School of Biological Sciences, Faculty of Science, The University of Adelaide, Adelaide, SA 5005, Australia; korjent.vandijk@adelaide.edu.au (K.-j.v.D.); bob.hill@adelaide.edu.au (R.S.H.); michelle.waycott@adelaide.edu.au (M.W.); 2 State Herbarium of South Australia, Adelaide, SA 5000, Australia; ed.biffin@adelaide.edu.au; 3 CAS Key Laboratory for Plant Diversity and Biogeography of East Asia, Kunming Institute of Botany, Chinese Academy of Sciences, Kunming 650204, China; liujie@mail.kib.ac.cn; 4 Germplasm Bank of Wild Species, Kunming Institute of Botany, Chinese Academy of Sciences, Kunming 650204, China

**Keywords:** conifers, gymnosperms, target capture sequencing, probe design, phylogenomics

## Abstract

**Simple Summary:**

Conifers are vital for both ecological and economic reasons, offering valuable insights into land plant evolution. Molecular phylogenetics plays a significant role in studying evolution, but research on conifers using large-scale data from multiple nuclear genes has been limited. Target enrichment sequencing has emerged as a crucial method in phylogenomic studies. However, a specific bait set for conifers is missing. The REMcon probe set targets around 100 single-copy nuclear loci for family- and species-level phylogenetic studies of conifers. High target recovery and read coverage were observed for the REMcon when tested on 69 species, including conifers and other gymnosperm taxa. Phylogenetic analysis based on the DNA sequences generated from REMcon recovered the existing understanding of conifer relationships. The REMcon bait set will be beneficial in generating large-scale nuclear data consistently for any conifer lineage.

**Abstract:**

Conifers are an ecologically and economically important seed plant group that can provide significant insights into the evolution of land plants. Molecular phylogenetics has developed as an important approach in evolutionary studies, although there have been relatively few studies of conifers that employ large-scale data sourced from multiple nuclear genes. Target enrichment sequencing (target capture, exon capture, or Hyb-Seq) has developed as a key approach in modern phylogenomic studies. However, until now, there has been no bait set that specifically targets the entire conifer clade. REMcon is a target sequence capture probe set intended for family- and species-level phylogenetic studies of conifers that target c. 100 single-copy nuclear loci. We tested the REMcon probe set using 69 species, including 44 conifer genera across six families and four other gymnosperm taxa, to evaluate the efficiency of target capture to efficiently generate comparable DNA sequence data across conifers. The recovery of target loci was high, with, on average, 94% of the targeted regions recovered across samples with high read coverage. A phylogenetic analysis of these data produced a well-supported topology that is consistent with the current understanding of relationships among conifers. The REMcon bait set will be useful in generating relatively large-scale nuclear data sets consistently for any conifer lineage.

## 1. Introduction

Conifers are the largest extant group among gymnosperms, with more than 722 species in 72 genera and 7 families, e.g., Araucariaceae, Cupressaceae, Pinaceae, Podocarpaceae, Sciadopityaceae, Cephalotaxaceae, and Taxaceae [1,2,3]. They are of great economic, ecological, and evolutionary significance, comprising approximately 39% of the world’s forests, and have a fossil record spanning more than 300 million years [4,5,6,7]. The complete understanding of conifer diversity, trait evolutions, genetic structure, and evolutionary history is still poorly explored [3,5,6]. Molecular phylogenetic studies play an important role in understanding the mode and tempo of evolution amongst conifers, but to date, most studies have applied a limited range of markers, principally a small number of chloroplast loci plus nuclear ribosomal DNA regions typically generated by direct amplicon sequencing (e.g., [5,8,9,10,11,12]). These studies have leveraged DNA direct amplicon sequencing data generated from these loci for phylogenetic and evolutionary analyses. Further complicating the study of molecular evolution in this major land plant lineage is the large genome size and overall complexity of conifer genomes [13,14], leaving a notable gap in the exploration of conifers using large-scale data. Target enrichment by hybridization capture (e.g., hyb-seq; Weitemier et al. 2014) [15] provides an efficient and cost-effective approach for generating DNA sequence data for a large number of single and low-copy nuclear gene regions across multiple samples.

Target enrichment approaches (File S1) have become the method of choice for many systematics, phylogenetic, and evolutionary studies in plants [16,17,18,19,20,21], in part fostered by the availability of ‘universal’ probe sets that can recover a common set of genes across broad evolutionary timescales. These include angiosperm-specific probe sets that target hundreds of nuclear genes [17,22] and one recently developed to enrich more than 400 nuclear genes across flagellate land plants [18]. While there are bait kits developed specifically for specific conifer families (e.g., Pinaceae; [23]), we are not aware of a conifer-specific bait set that targets the entire clade.

Here we present a new molecular toolkit (REMcon) which, based upon published transcriptomic (The 1000 Plant Transcriptomes Initiative, 1KP; [24]) and genomic data [25,26], uses RNA baits to target approximately 100 low-copy nuclear genes across conifers. In the present study, we demonstrate the universality and application of these new molecular tools for reconstructing phylogenetic relationships among conifers based on a broad sample of gymnosperm taxa. The approach typically recovers conservative coding regions plus more variable non-coding regions that flank the exons and has application for evolutionary analyses among closely related species, as we will demonstrate in an upcoming study of the *Podocarpus* ‘Australis’ clade (Khan et al., in prep) of family Podocarpaceae [6,27,28].

## 2. Materials and Methods

### 2.1. Probe Design

Target enrichment probes were designed using genes selected from Duarte et al. (2010) [29], who identified a set of orthologous low-copy nuclear genes shared across angiosperms (*Arabidopsis*, *Populus*, *Vitis* and *Oryza)*. For each of the selected genes, we extracted the putatively orthologous coding sequence (CDS) from the spruce (*Picea abies*, Pinaceae: https://plantgenie.org/, accessed on 4 April 2021) and western red cedar (*Thuja plicata*; https://phytozome-next.jgi.doe.gov/info/Tplicata_v3_1, accessed on 14 May 2021) genomes. These were used to retrieve putatively homologous transcript sequences for conifers from 1KP (https://www.onekp.com, accessed on 22 April 2021) using the China National GenBank (https://db.cngb.org, accessed on 12 April 2021) BLAST portal and the following settings: Discontiguous Mega-Blast, expect value = 10, maximum target sequences = 1000, selected organisms = Pinidae (taxid: 3313).

The sequences retrieved from the BLAST search for each target gene were downloaded and made into a BLAST database in Geneious (Kearse et al. 2012; https://www.geneious.com, accessed on 1 August 2021) [30]. We queried each BLAST database using the *Thuja plicata* gene family member with exon annotations manually added and the following settings: Discontiguous Mega-Blast, expect value = 10, maximum target sequences = 1200, results = Hit Table, retrieve = Matching Region with Annotation. We then extracted all sequences matching one or more exon annotations in *P*. *abies* with the caveat that the exon was >180 bp in length to allow for bait tiling. The extracted sequences were clustered using CD-HIT-EST ([31,32,33]; http://weizhong-lab.ucsd.edu/cdhit_suite, accessed on 4 August 2021) with a sequence identity cut-off fraction of 0.88 (see [34,35]) and a length similarity fraction of 0.2, and one representative sequence (the longest) per cluster was selected. A total of 1,124 representative sequences (mean length 1051 nucleotides, range 181–4416 nt) covering exons from 100 putative low-copy nuclear genes (Table 1; Table S1) were used for bait design with 120-nt baits and ~2× flexible tiling density for a total of 17,982 baits (see baits-Spruce [14853] for the nucleotide sequences of the baits). Bait design and synthesis were performed by Daicel Arbor Biosciences (formerly MYcroarray; Ann Arbor, MI, USA) in the generation of the myBaits Custom DNA-Seq kit™ Ann Arbor, MI, USA used for target enrichment-based next-generation sequencing.

### 2.2. Taxon Selection

A total of 44 conifer genera representing six families, three species of Cycadales, and Gingko biloba (69 taxa) were included to evaluate the efficiency of target capture across conifers and more widely among gymnosperms. Most plant specimens were freshly collected from the living collections held at the Botanic Gardens of South Australia and dried in silica gel, and some were sampled from preserved specimens held at the State Herbarium of South Australia (Table S2).

### 2.3. DNA Extraction, Library Preparation, Hybrid Capture and Sequencing

For DNA extractions, about 15 mg of silica gel dried leaf material per sample was used, and homogenized in a Omni ruptor (Omni International, Kennesaw, GA, USA) using ceramic beads. DNA was extracted using the Qiagen Plant Mini kit, QIAGEN, Germantown, MD, USA and normalized 2 ng/uL before proceeding to library preparation, which follows the steps outlined in Waycott et al. [36] for their nuclear bait set. Hybrid capture was performed following the manual provided by myBaits with a hybridization temperature of 65 °C and 150 bp paired-end sequencing was performed at the Australian Genome Research Facility (AGRF), Melbourne, Australia on an Illumina NovaSeq S1 flow cell.

### 2.4. Bioinformatics Analyses

High-throughput paired-end reads were de-multiplexed and quality trimmed (using a Phred score threshold of 20) using CLC Genomics Workbench (v. 20; https://www.qiagenbioinformatics.com/, accessed on 22 April 2022). The Sequence Capture Processor pipeline SECAPR v 2.2.3: Andermann et al. [37], http://antonellilab.github.io/seqcap_processor/, accessed on 24 April 2022) was used to generate nuclear DNA sequence data sets from the trimmed reads. First, the reads from each sample were assembled de novo using SPAdes [38] with default kmer values. Contigs matching the reference *Thuja plicata* reference sequences (i.e., annotated exons, see above) were extracted from the de novo assemblies with LASTZ v. 1.04 [39] using a target length of 0.5 and a similarity fraction of 0.75 (i.e., 50% of the contig has to overlap with the target gene and be no less than 75% similar). The ‘keep paralogs’ flag was activated and deactivated to assess the extent of paralogy in the data. SECAPR identifies paralogs as multiple overlapping contigs matching a target sequence, keeping the longest contig if the ‘keep paralogs’ flag is activated. The extracted contigs were aligned per locus to produce multiple sequence alignments (MSA) using MAFFT [40]. The aligned contigs were subsequently used for a reference-based assembly using the BWA read mapper v.0.7.16a-r1181 [41], and the ‘sample specific’ flag, i.e., each sample is extracted from the alignment and mapped separately. Consensus sequences per sample from subsequent read mappings were again aligned using MAFFT to produce MSAs for each targeted gene region. The approach developed by Yang and Smith [42] and modified with containerization for target capture data (Jackson et al. [43]; https://github.com/chrisjackson-pellicle/Yang-and-Smith-paralogy-resolution, accessed on 29 April 2022) was used to resolve groups of orthologous sequences (orthology inference) from targeted gene regions. Following various filtering steps, the approach uses phylogenetic tree-based methods and the pruning of duplicated taxa from rooted phylogenies to resolve orthologous groups of sequences. In this study, de novo contigs for each sample from the SECAPR pipeline (above) were first imported into Geneious Prime (v. 2022.0.1; (https://www.geneious.com, accessed on 24 May 2022) and made into a Blast database. This database was queried using the extracted contigs from an outgroup (*Ginkgo biloba*) matching the targeted gene regions in *P*. *abies*. The contigs from *Ginkgo* were annotated with the CDS from *P*. *abies,* and the coding region(s) were queried against the Blast database using blast-n with a maximum expected value of 1e-10 and maximum hits set to 1000. The Blast output was filtered using a minimum coverage fraction (query coverage of at least 0.4) to remove poorly aligned and short sequence fragments [42]. The resulting contigs were then used as input into the Yang-and-Smith-paralogy-resolution pipeline [43]. We used the monophyletic outgroups (MO) method to identify ortholog groups using reference genes from *Gingko biloba* as the outgroup. For downstream analyses, we retained alignments with >10 individuals in order to reduce the influence of missing data in tree inference. The aligned ortholog groups were concatenated, and a phylogeny was generated using IQ-tree 2 [44] using model finder [45] to estimate the optimum partitioning scheme and partition-specific nucleotide substitution model (MFP+MERGE flag activated) and 1000 ultrafast bootstrap [46] replicates to assess branch support.

## 3. Results and Discussion

**Figure 1:**
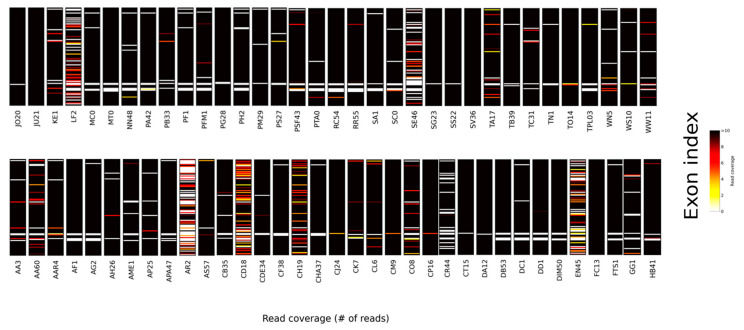
Heatmap showing gene recovery and read depth across samples. The gene recovery includes samples that were flagged as potentially paralogous using the SECAPR pipeline. Sample abbreviations as in Table S1.

The retrieval of target loci across the conifers was high, with, on average, 94% of the targeted gene regions recovered per sample (range 53–100), and 27 loci were recovered across all samples (Table 2). For the recovered loci, read coverage (read depth/position, averaged across loci) was also high, averaging c.146 across the included taxa with a maximum of 622 in *Chamaecyparis pisifera* and a minimum of c. 6 in *Agathis robusta* (Figure 1—coverage heatmap). In general, the recovery of genes across the six conifer families was relatively consistent, and with the exception of Araucariaceae, the mean number of loci captured per family exceeds 90 (Table 2). The lower mean value for Araucariaceae (87 loci) is influenced by the poor recovery for *Agathis robusta* (57 loci), which is likely a consequence of DNA quality and/or issues with the library construction, given that target recovery amongst close relatives (e.g., *Agathis microstachya,* 94 loci) was high (Figure 1, Table S2). There was a lower recovery of target genes for the non-coniferous gymnosperm species (c. 80% of genes recovered across the four samples), although this is not unexpected given that these were not specifically targeted in the probe design. Furthermore, the identity of the target sequences (here, sourced from *Thuja*) could influence locus recovery with increasing evolutionary distance, and an approach similar to McLay et al. [47] may be valuable in increasing target locus recovery from specific lineages.

**Figure 2:**
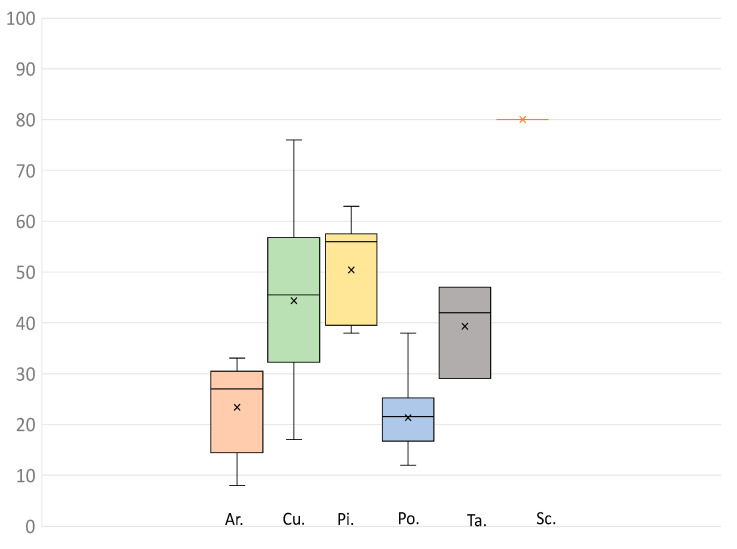
Box and whisker plots showing the recovery of paralogs (number of paralogous genes/sample) by family. Abbreviations: Ar., Araucariaceae; Cu., Cupressaceae; Pi., Pinaceae; Po., Podocarpaceae; Ta., Taxaceae; Sc., Sciadopityaceae. X = Mean, Middle horizontal bar = Median, and the lower bounds of the box are the 75 and 25 quartiles.

Overall, there was a large number of putatively paralogous gene copies recovered at most loci (c. 36% of loci/sample, averaged across all samples) but this was highly variable among taxa (*Prumnopitys andina*, Podocarpaceae: c. 13% of recovered loci; *Sciadopitys verticillata,* Sciadopityaceae: 80% of recovered loci) (Figure 2; Table S2). The extent of paralogy might reflect the generally large genome size of conifers, which is also highly variable, with at least an order of magnitude difference between the smallest and the largest conifer genomes [14]. Polyploidy is a major driver of genome size evolution amongst angiosperms, although until recently [48,49], this phenomenon was thought to be relatively rare among conifers (e.g., [13,50,51]). In addition, conifer genome size evolution has been attributed to other factors, such as a high copy number of long transposable elements (e.g., [25]). The distribution of paralogs in our data supports the view that genome size, per se, is only partly related to the frequency of duplicated genes. For instance, Podocarpaceae has the smallest average genome size [14] and the smallest proportion of paralogs in our data. On the other hand, Pinaceae generally have large genomes, and we found a large proportion of putative paralogs among samples from this family. However, the relatively large genome size among Araucariaceae is not strongly associated with a high number of paralogous genes in our data (Figure 2), suggesting that factors other than gene duplication (e.g., transposable elements, larger introns, and abundant pseudogenes; [13,25,48] are also important drivers of genome size evolution.

**Figure 3:**
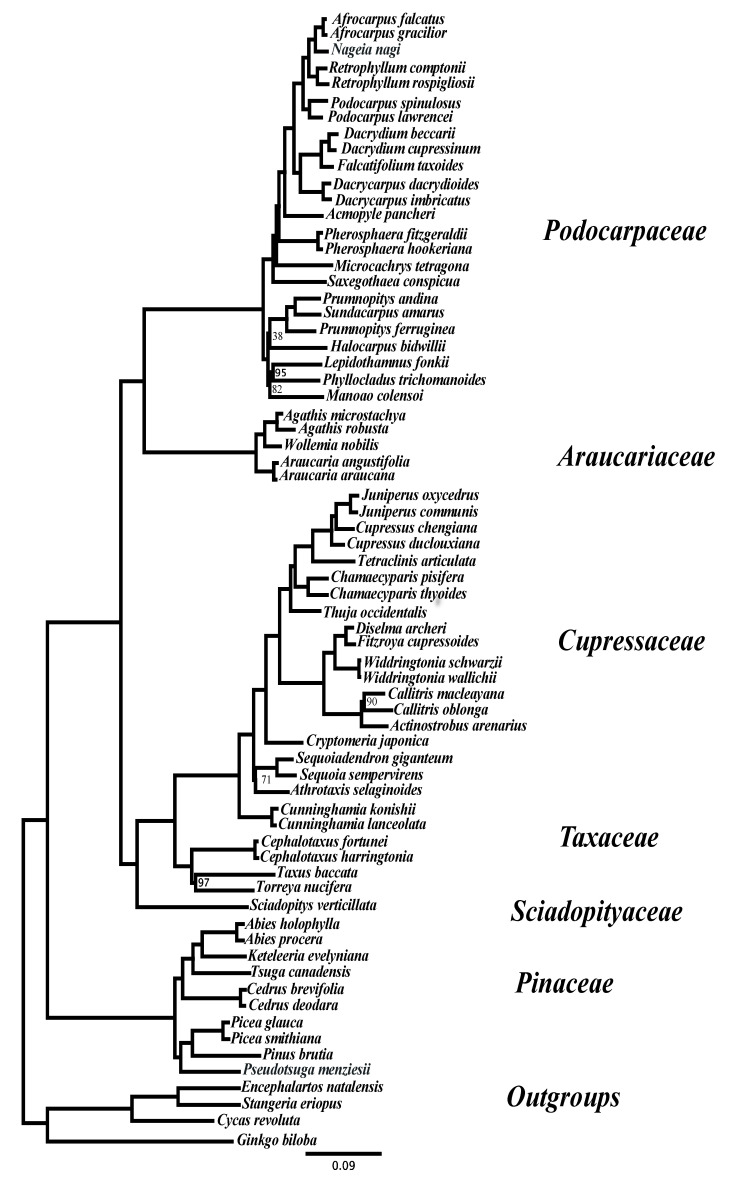
Maximum likelihood phylogeny estimated from the concatenated MO ortholog gene alignments. All branches have maximum bootstrap support unless indicated adjacent to the branch. Sample abbreviations as in Table S1.

Of the 100 targeted gene regions, 90 were recovered for *Gingko*, and these were included in the paralogy resolution analyses. Orthology inference recovered 98 MO ortholog groups and 95 with more than 10 samples included, which were retained for phylogenetic inference. The concatenated length of the 95 loci was an average of c. 48,770 bp with an aligned length of c. 74,179 bp and approximately 34% missing values. The average aligned length of the individual loci was c. 780 bp and ranged from 440–1691 bp. The concatenated alignment includes 47,469 (c. 64%) variable positions, of which 36,350 (c. 49%) are parsimony informative and 44,818 (c. 60%) variable and 34,411 (c. 46%) parsimony informative characters within the conifer clade. The maximum likelihood topology inferred from these data is shown in Figure 3. Of the 65 clades recovered, only 7 have a bootstrap support value < 100, and of these, only 3 received less than 80% support (Figure 3). The inferred topology is generally in agreement with our current understanding of conifer relationships (e.g., [2,3,5]), while the poorly supported nodes are associated with short branches and may be inherently difficult to resolve (e.g., [52,53,54]). For example, the relationships within the Prumnopityoid clade of Podocarpaceae, and in particular the placement of *Halocarpus,* were found to be unstable in the recent analyses of Chen et al. [55] using a large transcriptome data set of c. 1000 nuclear and c. 40 chloroplast gene regions and is poorly resolved here (Figure 3).

## 4. Conclusions

In conclusion, we present a conifer-specific hybrid-capture bait set that has been shown to perform well in terms of the consistency of locus recovery across a broad range of gymnosperms, and these data can be applied to credibly resolve deep phylogenetic relationships within the conifer clade. As part of ongoing studies (Khan et al. in prep) [6,27,28], we have found the REMcon bait set to be similarly successful in resolving relationships among closely related species groups within Podocarpaceae. The REMcon bait set offers an efficient and relatively cost-effective approach that fills an important gap in conifer and gymnosperm phylogenomics. This hybrid-capture bait set has exciting future applications, including the resolution of complex phylogenetic relationships, population, and comparative genomics, providing valuable insights into the evolution and conservation of conifers and other gymnosperms.

## Figures and Tables

**Table 1:** Targeted nuclear gene regions and the length of the probe sequences.

S#	*Picea abies* Gene Name	*Arabidopsis thaliana* Putative Homolog	Length of the Probe Sequences
1	MA_10437158	AT5G06430	195
2	MA_10437143	AT1G12370	480
3	MA_10437077	AT5G02250	621
4	MA_10437070	AT5G10920	720
5	MA_10436603	AT1G03750	945
6	MA_10436489	AT4G37510	878
7	MA_10435966	AT4G38890	822
8	MA_10435879	AT2G33630	613
9	MA_10435851	AT5G04520	510
10	MA_10435433	AT2G44760	426
11	MA_10435005	AT2G40570	787
12	MA_10434812	AT1G36310	1088
13	MA_10434753	AT1G49380	539
14	MA_10433768	AT2G31955	942
15	MA_10433107	AT5G64150	825
16	MA_10432498	AT1G74640	453
17	MA_10431375	AT2G24830	321
18	MA_10430781	AT4G35910	432
19	MA_10429426	AT1G30070	240
20	MA_10428930	AT1G15390	256
21	MA_10428614	AT2G34640	259
22	MA_10428345	AT1G57770.1	315
23	MA_10428134	AT2G04560	273
24	MA_10427767	AT1G21370	291
25	MA_10427729	AT5G67530	1224
26	MA_10427590	AT1G17160	480
27	MA_10427203	AT2G36740	543
28	MA_10426631	AT4G36390	1533
29	MA_10426581	AT2G33450	231
30	MA_10426376	AT2G38270	504
31	MA_9578808	AT4G18372	387
32	MA_9514062	AT5G20220	315
33	MA_9503281	AT1G48175	257
34	MA_8815984	AT2G346401	693
35	MA_8715484	AT4G38020	501
36	MA_8687206	AT4G26980	408
37	MA_8286794	AT3G17170	342
38	MA_8140147	AT2G28605	480
39	MA_7890741	AT2G44660	783
40	MA_5587080	AT4G20060	447
41	MA_957334	AT1G05055	462
42	MA_945784	AT5G06410	380
43	MA_939779	AT4G27390	468
44	MA_938037	AT5G49570	580
45	MA_894439_	AT2G30100	1306
46	MA_824260	AT4G28020	441
47	MA_762004	AT1G28560	675
48	MA_759516	AT5G08720	461
49	MA_749379	AT4G11980	201
50	MA_587488	AT4G01040	377
51	MA_546546	AT4G17760	252
52	MA_537299	AT5G54840	264
53	MA_458270	AT5G06830	690
54	MA_388031	AT2G20330	486
55	MA_341112	AT5G11980	276
56	MA_332596	AT2G34460	333
57	MA_314789	AT1G56345.1	603
58	MA_261436	AT4G33030	1290
59	MA_253636	AT3G51050	768
60	MA_225872	AT5G14260	456
61	MA_224167	AT2G20790	900
62	MA_199851	AT3G01660	350
63	MA_196209	AT4G36530	273
64	MA_187402	AT4G31460	471
65	MA_173127	AT4G28740	548
66	MA_159115	AT2G27600	1191
67	MA_159115	AT4G27600	1056
68	MA_127668	AT3G15290	465
69	MA_123340	AT2G19870	1137
70	MA_121485	AT1G02410	749
71	MA_121026	AT1G08460	570
72	MA_106933	AT2G266801	636
73	MA_104872	AT3G26580	507
74	MA_99242	AT4G29070	412
75	MA_98424	AT1G07130	558
76	MA_95157	AT5G09820	292
77	MA_83545	AT5G65860	514
78	MA_78599	AT2G40760	252
79	MA_73742	AT2G21840	939
80	MA_73742	AT1G21840	939
81	MA_67861	AT2G26680	369
82	MA_66902	AT2G36145	234
83	MA_66902	AT2G34145	234
84	MA_63465	AT3G24315	290
85	MA_61548	AT1G65030	681
86	MA_55048	AT5G19130	858
87	MA_43083	AT5G48330	717
88	MA_41847	AT3G03790	303
89	MA_35149	AT3G02300	431
90	MA_34295	AT1G43580	378
91	MA_30194	AT5G16210	369
92	MA_29076	AT3G57910	513
93	MA_26068	AT2G37560	414
94	MA_25177	AT1G07970	472
95	MA_24252	AT4G24090	600
96	MA_19954	AT2G02590	414
97	MA_11407	AT3G47860	312
98	MA_10909	AT2G04270	318
99	MA_6888	AT3G24080	2286
100	MA_4586	AT2G22650	303

**Table 2:** Recovery of targeted gene regions, including potentially paralogous loci, across conifer families and four non-conifer gymnosperms. Locus recovery is averaged across samples (*n*), and the minimum and maximum recovery per sample is indicated.

Families	*N*	Average Locus Recovery	Min	Max
Araucariaceae	5	85	53	97
Cupressaceae	22	98	89	100
Pinaceae	11	96	95	90
Podocarpaceae	26	93	76	97
Sciadopityaceae	1	95	-	-
Taxaceae	3	97	96	97
Non conifer Gymnosperms	4	81	75	92

## Data Availability

Data are contained within the article or Supplementary Materials.

## Supplementary Materials

The following supporting information can be downloaded at https://www.mdpi.com/article/10.3390/biology13060361/s1, Table S1: Details of targeted genes; Table S2: Sample details; Table S3: Recovery of targeted gene regions across conifer familes and four non conifer gymnosperms. Locus recovery is averaged across samples (n) and the minimum and maximum recovery per seample is indicated. Figure S1: Paralogs/Family; File S1: HybCap76 NEBnext Podocarp03 half plate.
