# Supplementary material for: Development of a Target Enrichment Probe Set for Conifer (REMcon)
Source: Biology (Basel). 2024 May 22;13(6):361. doi: 10.3390/biology13060361 (PMC11200496; doi:10.3390/biology13060361)
Supplement: Supplementary file 1 [file biology-13-00361-s001.zip › biology-2960267-supplementary/Sup data/File S1 HybCap76 NEBnext Podocarp03 half plate .pdf]

Protocol for library preparation for hybridisation capture

DNA normalisation protocol for 96well plate on EPmotion

- **Plate to List conversion xlsx:**
  - Open sheet and paste (paste special: values) DNA quants in the 96 well plate layout format in **sheet 1**
  - Copy resulting column
  - Should be in format A1,B1,C1 etc. but need to get to format A1,A2,A3

- **Plate to List conversion xlsx:**
  - Go to **sheet 2** (Flipper) and paste column from sheet 1 (paste special: values) onto **column J** (ignore column H until the end)
  - **Column J** is in the order A1,B1,C1 – check whether this matches **column G** if not re order column G to A1,B1,C1 by sorting by **column F**
  - Now both **column G** and **column J** are in the order A1,B1,C1
  - Highlight all **columns E-J** and re order to the format A1,A2,A3 by sorting by **column E**.
  - Paste **column J** into **column H**

- **Normalisation Calculator_TEMPLATE_7.2018.xlsx:**
  - Open sheet and paste **column H** FROM PREVIOUS onto **column J** (first sheet)
  - Check volume is 50 ul (F4) & final concentration is 1ng/uL (or 2ng/uL) (F6)
  - Create separate CSV files for water and DNA quantities under **column N** (note: there can only be a ‘y’ entered at one of the two boxes at one time only). The data will be automatically generated in sheet 3.
  - Start with DNA csv file first, check the values are correct. Copy data in rows A-H and check that everything is correct. If any wells contain 0 ng / ul, change the value to "50". paste into a new workbook. Export as a csv file for windows ("Windows CRLF" in Mac; "CSV (MS-DOS) in Windows)
  - Then create the file for water but make sure you **change the source (column C) to 1** instead of the wells (A1) and delete any rows that have “invalid tool”/ are 0 (no water needed) or 50 (derived from wells with 0 ng / ul DNA) for volume. Check the values are correct. Copy data in rows A-H and paste into a new workbook. Export as a csv file for windows ("Windows CRLF" in Mac; "CSV (MS-DOS) in Windows)
  - Open both the DNA file and Water file in a text editor such as BBEdit (Mac) or Notepad++ (Windows).
  - In each file, using the "search" function, replace all: replace "/n" with ", /n" to add an extra comma to each row (necessary for EpMotion to accept the file correctly). The /n represents the blank space at the end of each row. It finds the blank space and appends a comma, then re-adds the blank space after it.
  - Save each file.
  - You should have two csv files, one for DNA and one for water, Copy the files to a clean USB to take into the lab.

- **Programming robot**
    - Initialise robot computer, start Eppendorf program -> Load files -> Korjent -> Dillutions -> Normalization Template BLANK
    - Select the second step (i.e. transfer of water to chimney plate) and import water csv file.
    - Ensure filter tips is selected. Choose the second pipette option and leave "change tips" UNSELECTED
    - Water steps should load
    - Scroll right to the bottom and select the final step (i.e. transfer from AGRF plate to Chimney plate and import the DNA csv file.
    - Ensure filter tips is selected. Choose the second pipette option and ENSURE "change tips" IS SELECTED / CHECKED
    - Set volume of all samples in DNA plate to 100uL – you will be prompted to do this (set all button in bottom left corner)

- **Preparing plates**

    - Place DNA / AGRF tray in the centrifuge and ensure the correct balance is used.
    - Spin for 1 min.
    - Label the chimney plate (no skirt, clear letters/numbers) with sample name, "normalisation", and the date. You can also colour the letters / numbers on the underside of the plate with a texta to make the sonication / pipetting step easier.

- **Setting up robot**

    - Place 50uL filter tips in positions marked on computer. Ensure that the grey tip holder is inserted so that the circles are on the left side, otherwise it will not recognise the tips.
    - Place molecular grade water in first tray position.
    - Place chimney plate to the left of the water as marked on computer.
    - Carefully remove DNA cover and place to the right of trays as marked by the computer
    - Do a check (tick symbol)
    - Start run (play symbol)
    - Once run is finished cap the chimney plate with bubble cap strips and place in fridge or freezer (doesn't matter as next step is to fragment the DNA anyway)
    - Place aluminum cover over DNA extraction plate with roller and put in freezer
    - Clean up workspace

Tips :
Can check csv on text editing software (e.g. Text Wrangler)
Check csv file is saved in Windows (CRLF) format in Text Wrangler (bottom row of screen)
If you get logged out username: Korjent , password: marieke22

Sonicating protocol (shearing to size distribution 400-600bp)

- Fill sonicating bath with distilled water past minimum mark, turn on so that it can cool to 4 degrees (takes ~ 40 min)
- Pipette normalised DNA from chimney plate (previous section) into sonicating tubes (all DNA must be transferred)
- Replace lid on chimney plate after each column is moved as the DNA will be placed back in here and do not want to leave exposed
- Once all wells have been transferred to sonicating tubes take to Darling Building level 3
- Fill sonicating bath with deionised water to specified level (Plastic container)
- Place 12 sonicating tubes into correct holder (tubes only fit in slightly sideways)
- Turn on sonicating machine and set Run cycle to 15 Sec on, 90 Sec off, repeating 6 times. (amount of cycles might vary depending on age, old samples might need fewer cycles).
- Press "ok" and sonicating will begin once the lid is closed
- After run cycle leave the samples in the water bath for 20 min to cool down (it is recommended to bring work to do in Darling so you are not travelling back and forth between Braggs)
- Repeat until all 96 (or more) samples are sonicated
- Transfer all sample in sonicating tube correctly back into Chimney plate with strip caps on

Can stop at this point place chimney plate in fridge but don't leave too long before doing library prep (couple of days)

Library Prep (to be done all in one day)

Reference information

The goal here is to prepare DNA libraries having unique Illumina TruSeq library adapters on a per-library basis. We will enrich these libraries using solution hybridization, also known as Target Enrichment or Hybrid Capture (e.g. see Gnirke *et al.* 2009 and Blumenstiel *et al.* 2010).

Original source

Library prep NEBNext® UltraTM II DNA Library Prep with Sample Purification Beads Kit and Hybrid Capture protocol with MyBait RNA Hybrid Capture Kit

Bibliography

Blumenstiel B, Cibulskis K, Fisher S, DeFelice M, Barry A, Fennell T, Abreu J, Minie B, Costello M, Young G, Maquire J, Kernytsky A, Melnikov A, Rogov P, Gnirke A, Gabriel S: **Targeted exon sequencing by in-solution hybrid selection.** *Curr Protoc Hum Genet* 2010, **Chapter 18**:Unit 18.4.

Faircloth BC, Glenn TC: **Not all sequence tags are created equal: designing and validating sequence identification tags robust to indels**. PLoS One 2012 7: e42543.

Fisher S, Barry A, Abreu J, Minie B, Nolan J, Delorey TM, Young G, Fennell TJ, Allen A, Ambrogio L, Berlin AM, Blumenstiel B, Cibulskis K, Friedrich D, Johnson R, Juhn F, Reilly B, Shammas R, Stalker J, Sykes SM, Thompson J, Walsh J, Zimmer A, Zwirko Z, Gabriel S, Nicol R, Nusbaum C: **A scalable, fully automated process for construction of sequence-ready human exome targeted capture libraries.** *Genome Biol* 2011, **12**:R1.

Gnirke A, Melnikov A, Maguire J, Rogov P, LeProust EM, Brockman W, Fennell T, Giannoukos G, Fisher S, Russ C, Gabriel S, Jaffe DB, Lander ES, Nusbaum C: **Solution hybrid selection with ultra-long oligonucleotides for massively parallel targeted sequencing**. *Nature Biotechnology* 2009, **27**:182–189.

NEBNext® UltraTM II DNA Library Prep with Sample Purification Beads manual

MyBait manual at http://www.mycroarray.com/mybaits/manuals.html

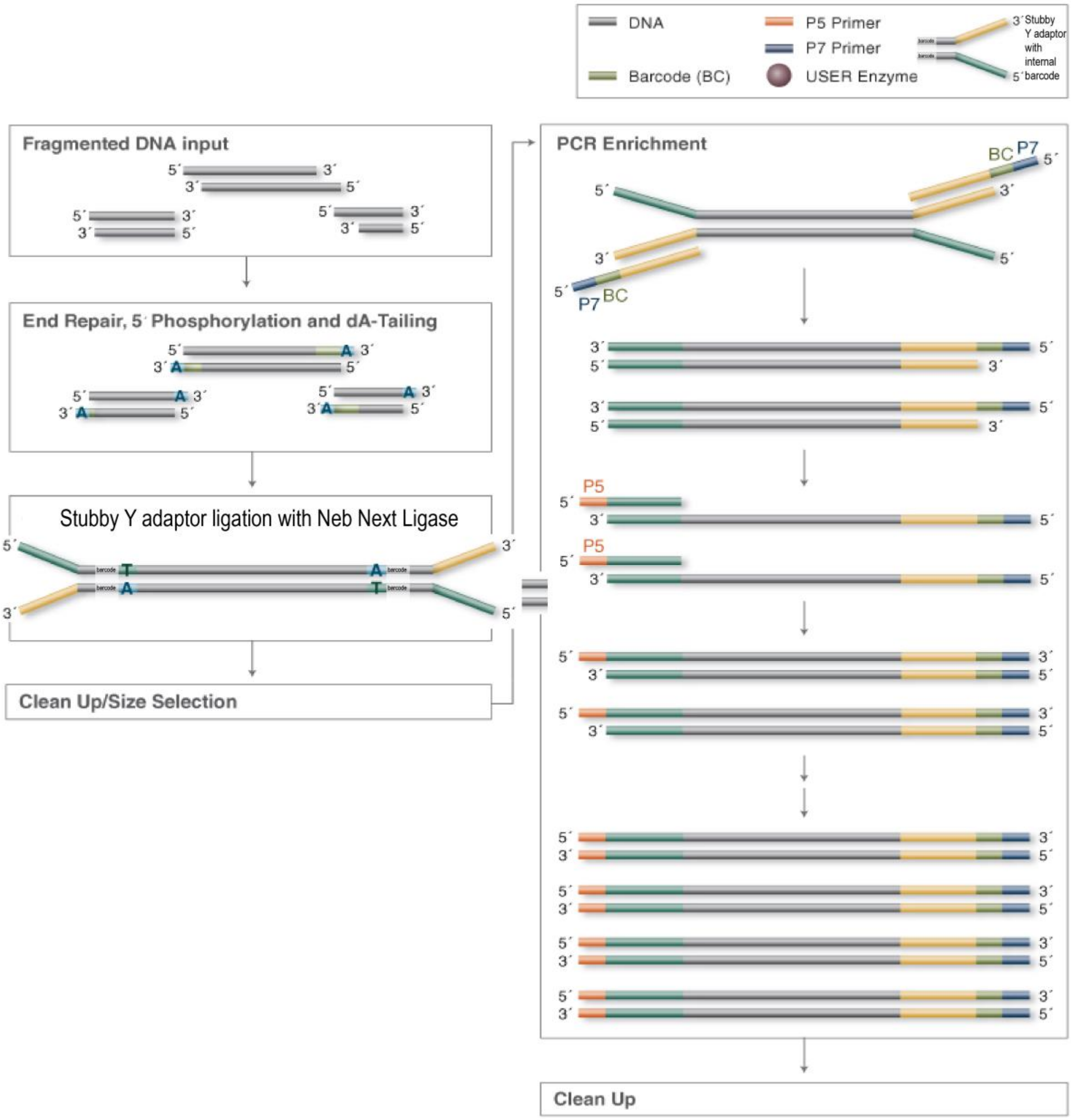

Figure 1 Nebnext II ultra library prep procedure with barcoded stubby Y-adapters and indexed primers.

Y- Adapters are prepared by Kor and stored in fridge

If you are using custom sequencing adapters [i.e., not the standard Illumina TruSeq adapters] but the study Y adapter developed in the lab with internal 8 nucleotide barcodes (Faircloth and Glenn 2012), and you have ordered both P5 and P7 oligos from IDT, you need to make the tagged adapters. This means combining the P5 tag### with the P7 complement tag### and annealing those to make double-stranded Y-adapters. You need to do this for all adapters you will use, and it is best/easiest to do this in a PCR plate.

1. Assemble the following components to make Annealing Buffer:

   9.9 mL 1X TE (10 mM Tris pH 8.0, 1 mM EDTA)
   0.1 mL 5 M NaCl

   10 mL Total

2. Using the Oligo Buffer, assemble the following components for to create the adapters:

   25 uL Oligo Buffer (Or labelled annealing buffer)
   12.5 uL 100 uM P5 Adapter
   12.5 uL 100 uM P7 Adapter (with or without index)

   50 uL Total 25uM Adapter

3. Anneal:

   95 C for 1 minute
   ⇨ -0.1 C/sec for 800 seconds (13.33 minutes)
   ⇨ 14 C hold

4. Store adapters at -20 C. (you might have to dilute this further to 2.5uM for library prep)

Y adapters repeat themselves every 48 samples and the same combination is used for every plate

Preparation of Adapter Solution

1. Prepare an Adapter Solution by diluting adapter stocks in Annealing Buffer. *We generally use either 25uM or 2.5uM adapter solution.*
2. Table 2. Recommended adapter concentration for varying starting amounts of DNA input.

Annealing Buffer (40mL)
- 40ul Tris HCl (1M)
- 8ul EDTA (0.5 M)
- 400 ul NaCl (5M)
- 39.5 ml H2O (Molecular)

Table 1 Recommended adapter concentration for varying starting amounts of DNA input.

DNA input amount	**Adapters concentration**	**Adapter : Insert Molar Ratio***
1 ug	30uM	15:1
100ng	10uM	50:1
10ng	**4uM**	**200:1**
1ng	1.5uM	750:1

*Adapter:insert molar ratio calculations are based on DNA fragments of 150 bp. Users are advised to use this table as guideline to optimize the adapter:insert molar ratio for DNA Input values different from the ones shown in this table.

For this reaction **12.5uM** adapters will give a final concentration of 0.8 uM, this is 1:32 Volume (25uM Adapter: Total Volume) ration in Mix. If DNA is low and inserts longer using 2.5uM Adapter might be better, resulting in a 1:160 dilution.

Sample details

**Run code**	**Well**	**Sample code**	**Region**	**Population**	**Conc. Ng/ull**	**Final conc for Fragmentation (ng/ul)**
**Podocarp03**						
Podo_193	A01	Podo_193_Acmopyle_sahniana_Mount_Lofty_Botanic_Park_Living_Collection_ASA_51	0	Mount Lofty Botanic Park Living Collection	0.00	0.00
Podo_194	B01	Podo_194_Acmopyle_pancheri_Mount_Lofty_Botanic_Park_Living_Collection_APA_47	0	Mount Lofty Botanic Park Living Collection	0.00	0.00
Podo_195	C01	Podo_195_Athrotaxis_laxifolia_Mount_Lofty_Botanic_Park_Living_Collection_AL_58	0	Mount Lofty Botanic Park Living Collection	0.00	0.00
Podo_196	D01	Podo_196_Athrotaxis_selaginoides_Mount_Lofty_Botanic_Park_Living_Collection_AS_57	0	Mount Lofty Botanic Park Living Collection	0.00	0.00
Podo_197	E01	Podo_197_Dacrycarpus_imbricatus_Mount_Lofty_Botanic_Park_Living_Collection_DIM_50	0	Mount Lofty Botanic Park Living Collection	0.00	0.00
Podo_198	F01	Podo_198_Dacrydium_beccarii_Mount_Lofty_Botanic_Park_Living_Collection_DB_53	0	Mount Lofty Botanic Park Living Collection	0.00	0.00
Podo_199	G01	Podo_199_Falcatifolium_taxoides_Sp1_large_leaves_Mount_Lofty_Botanic_Park_Living_Collection_FT_S1	0	Mount Lofty Botanic Park Living Collection	0.00	0.00
Podo_200	H01	Podo_200_Falcatifolium_taxoides_Sp2_small_leaves_Mount_Lofty_Botanic_Park_Living_Collection_FT_S2	0	Mount Lofty Botanic Park Living Collection	0.00	0.00
Podo_201	A02	Podo_201_Retrophyllum_comptonii_Mount_Lofty_Botanic_Park_Living_Collection_RC_54	0	Mount Lofty Botanic Park Living Collection	0.00	0.00
Podo_202	B02	Podo_202_Retrophyllum_rospigliosii_Mount_Lofty_Botanic_Park_Living_Collection_RR_55	0	Mount Lofty Botanic Park Living Collection	0.00	0.00
Podo_203	C02	Podo_203_Abies_holophylla_Mount_Lofty_Botanic_Park_Living_Collection_AH_26	0	Mount Lofty Botanic Park Living Collection	0.00	0.00
Podo_204	D02	Podo_204_Abies_procera_Mount_Lofty_Botanic_Park_Living_Collection_AP_25	0	Mount Lofty Botanic Park Living Collection	0.00	0.00
Podo_205	E02	Podo_205_Agathis_australis_Mount_Lofty_Botanic_Park_Living_Collection_AG_1	0	Mount Lofty Botanic Park Living Collection	0.00	0.00
Podo_206	F02	Podo_206_Agathis_microstachya_Mount_Lofty_Botanic_Park_Living_Collection_AME1	0	Mount Lofty Botanic Park Living Collection	0.00	0.00
Podo_207	G02	Podo_207_Araucaria_araucana_Mount_Lofty_Botanic_Park_Living_Collection_AAR_4	0	Mount Lofty Botanic Park Living Collection	0.00	0.00
Podo_208	H02	Podo_208_Callitris_macleayana_Mount_Lofty_Botanic_Park_Living_Collection_CM_9	0	Mount Lofty Botanic Park Living Collection	0.00	0.00
Podo_209	A03	Podo_209_Cedrus_brevifolia_Mount_Lofty_Botanic_Park_Living_Collection_CB_35	0	Mount Lofty Botanic Park Living Collection	0.00	0.00
Podo_210	B03	Podo_210_Cedrus_deodara_Mount_Lofty_Botanic_Park_Living_Collection_CDE_34	0	Mount Lofty Botanic Park Living Collection	0.00	0.00
Podo_211	C03	Podo_211_Cephalotaxus_fortunei_Mount_Lofty_Botanic_Park_Living_Collection_CF_38	0	Mount Lofty Botanic Park Living Collection	0.00	0.00
Podo_212	D03	Podo_212_Cephalotaxus_harringtonia_Mount_Lofty_Botanic_Park_Living_Collection_CHA_37	0	Mount Lofty Botanic Park Living Collection	0.00	0.00
Podo_213	E03	Podo_213_Chamaecyparis_pisifera_Mount_Lofty_Botanic_Park_Living_Collection_CP_16	0	Mount Lofty Botanic Park Living Collection	0.00	0.00
Podo_214	F03	Podo_214_Chamaecyparis_thyoides_Mount_Lofty_Botanic_Park_Living_Collection_CT_15	0	Mount Lofty Botanic Park Living Collection	0.00	0.00
Podo_215	G03	Podo_215_Cryptomeria_japonica_Mount_Lofty_Botanic_Park_Living_Collection_CJ_24	0	Mount Lofty Botanic Park Living Collection	0.00	0.00
Podo_216	H03	Podo_216_Cycas_revoluta_Mount_Lofty_Botanic_Park_Living_Collection_CR_44	0	Mount Lofty Botanic Park Living Collection	0.00	0.00
Podo_217	A04	Podo_217_Diselma_archeri_Mount_Lofty_Botanic_Park_Living_Collection_DA_12	0	Mount Lofty Botanic Park Living Collection	0.00	0.00
Podo_218	B04	Podo_218_Fitzroya_cupressoides_Mount_Lofty_Botanic_Park_Living_Collection_FC_13	0	Mount Lofty Botanic Park Living Collection	0.00	0.00
Podo_219	C04	Podo_219_Ginkgo_biloba_Mount_Lofty_Botanic_Park_Living_Collection_GG_1	0	Mount Lofty Botanic Park Living Collection	0.00	0.00
Podo_220	D04	Podo_220_Juniperus_cummunis_Mount_Lofty_Botanic_Park_Living_Collection_JU_21	0	Mount Lofty Botanic Park Living Collection	0.00	0.00
Podo_221	E04	Podo_221_Juniperus_oxycedrus_Mount_Lofty_Botanic_Park_Living_Collection_JO_20	0	Mount Lofty Botanic Park Living Collection	0.00	0.00
Podo_222	F04	Podo_222_Keteleeria_evelyniana_Mount_Lofty_Botanic_Park_Living_Collection_ke_1	0	Mount Lofty Botanic Park Living Collection	0.00	0.00
Podo_223	G04	Podo_223_Picea_glauca_Mount_Lofty_Botanic_Park_Living_Collection_PG_28	0	Mount Lofty Botanic Park Living Collection	0.00	0.00
Podo_224	H04	Podo_224_Picea_smithiana_Mount_Lofty_Botanic_Park_Living_Collection_PS_27	0	Mount Lofty Botanic Park Living Collection	0.00	0.00

Podo_225	A05	Podo_225_Pinus_brutia_Mount_Lofty_Botanic_Park_Living_Collection_PB_33	0	Mount Lofty Botanic Park Living Collection	0.00	0.00
Podo_226	B05	Podo_226_Pinus_contorta_Mount_Lofty_Botanic_Park_Living_Collection_PC_32	0	Mount Lofty Botanic Park Living Collection	0.00	0.00
Podo_227	C05	Podo_227_Pseudotsuga_menziesii_Mount_Lofty_Botanic_Park_Living_Collection_PM_29	0	Mount Lofty Botanic Park Living Collection	0.00	0.00
Podo_228	D05	Podo_228_Pseudotsuga_sinensis_Mount_Lofty_Botanic_Park_Living_Collection_PS_30	0	Mount Lofty Botanic Park Living Collection	0.00	0.00
Podo_229	E05	Podo_229_Sciadopitys_verticillata_Mount_Lofty_Botanic_Park_Living_Collection_SV_36	0	Mount Lofty Botanic Park Living Collection	0.00	0.00
Podo_230	F05	Podo_230_Sequoia_sempervirens_Mount_Lofty_Botanic_Park_Living_Collection_SS_22	0	Mount Lofty Botanic Park Living Collection	0.00	0.00
Podo_231	G05	Podo_231_Sequoiadendron_giganteum_Mount_Lofty_Botanic_Park_Living_Collection_SG_23	0	Mount Lofty Botanic Park Living Collection	0.00	0.00
Podo_232	H05	Podo_232_Stangeria_eriopus_Mount_Lofty_Botanic_Park_Living_Collection_SE_46	0	Mount Lofty Botanic Park Living Collection	0.00	0.00
Podo_233	A06	Podo_233_Sundacarpus_amarus_Mount_Lofty_Botanic_Park_Living_Collection_SA_1	0	Mount Lofty Botanic Park Living Collection	0.00	0.00
Podo_234	B06	Podo_234_Taxus_baccata_Mount_Lofty_Botanic_Park_Living_Collection_TB_39	0	Mount Lofty Botanic Park Living Collection	0.00	0.00
Podo_235	C06	Podo_235_Thuja_occidentalis_Mount_Lofty_Botanic_Park_Living_Collection_TO_14	0	Mount Lofty Botanic Park Living Collection	0.00	0.00
Podo_236	D06	Podo_236_Torreya_nucifera_Mount_Lofty_Botanic_Park_Living_Collection_TN_1	0	Mount Lofty Botanic Park Living Collection	0.00	0.00
Podo_237	E06	Podo_237_Tsuga_canadensis_Mount_Lofty_Botanic_Park_Living_Collection_TC_31	0	Mount Lofty Botanic Park Living Collection	0.00	0.00
Podo_238	F06	Podo_238_Widdringtonia_schwarzii_Mount_Lofty_Botanic_Park_Living_Collection_WS_10	0	Mount Lofty Botanic Park Living Collection	0.00	0.00
Podo_239	G06	Podo_239_Widdringtonia_wallichii_Mount_Lofty_Botanic_Park_Living_Collection_WW_11	0	Mount Lofty Botanic Park Living Collection	0.00	0.00
Podo_240	H06	Podo_240_Wollemia_nobilis_Mount_Lofty_Botanic_Park_Living_Collection_WN_5	0	Mount Lofty Botanic Park Living Collection	0.00	0.00

Plate prep for robot

1. Manually dispense 16.7 ul of sonicated DNA from chimney plate into LowBind Eppendorf Twin Tec semi skirted plate, to use on robot. Use multichannel pipette.

End-Repair

This step repairs the ends of the DNA after sonication as there may be fragments that are partly broken. This step also phosphorylates the ends of the DNA.

1. Fill esky with ice from level 3 student labs (in the right-hand corner of the room) * if you cannot get in find someone in the office next door to let you in*
2. Remove ALL reagents from storage (-20 °C) and place on ice, including: **END PREP** reagents, **LIGATION MIX** reagents, and **MASTER MIX** reagents. but keep separate so as to not get confused. Briefly vortex and spin down each reagent before use.
3. Remove Y-adapters from the freezer to defrost (made previously by Kor) should be in a chimney-plate with strip caps.

4. Start EpMotion (robot) and load the protocol "**HybCap_to96_samples ER and ligation NEBnext**". Set up the robot as indicated on the computer or as shown below:

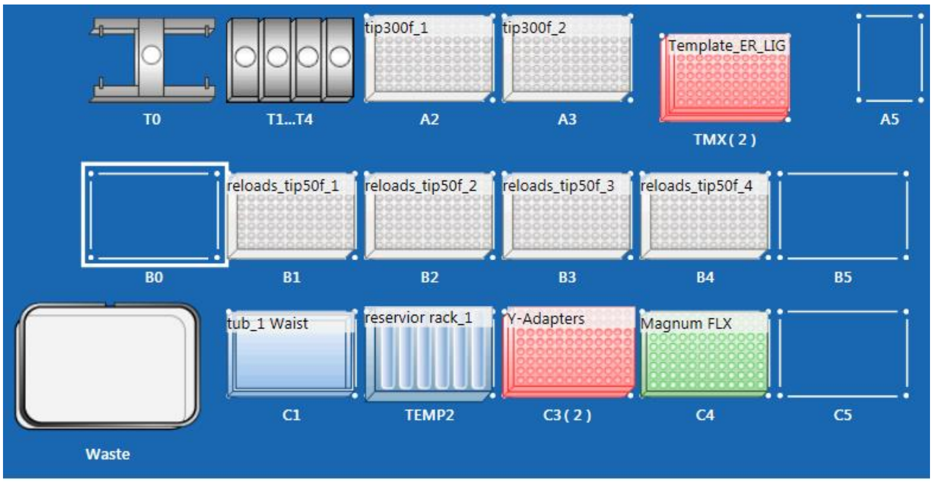

Figure 2 Epmotion deck layout for end repair and ligation protocol.

FRONT OF ROBOT

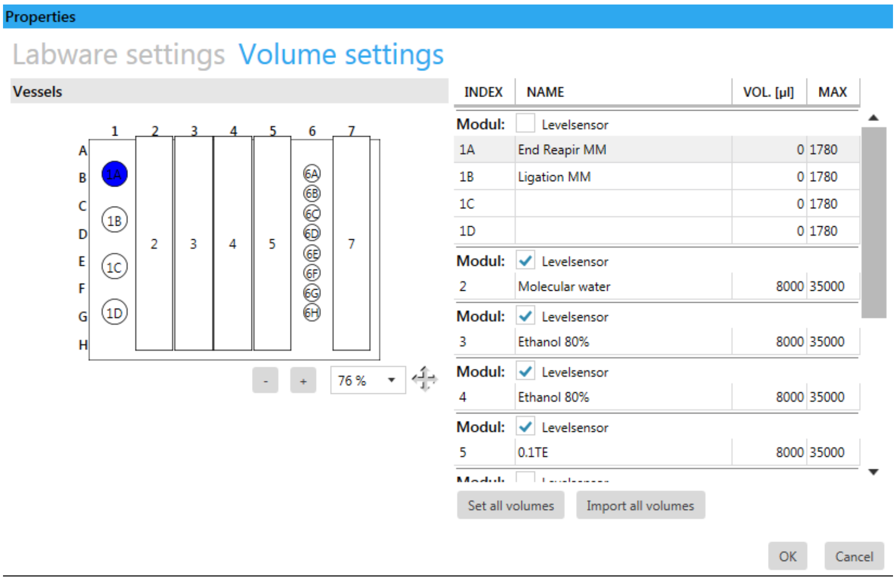

Figure 3 placement of tubes and tubs in ResRack (reservoir rack).

Note: Yellow 300uL pipette tips are placed in grey containers that need to be BLEACHED BEFORE EACH RUN!

*The ResRack (Reservoir Rack) should be set up as follows:

Column 1: 1.5 Eppendorf tubes (4 "larger tubes" thermoblock – for NEBnext reagents in freezer, to be kept on ice until needed)

Column 2: H_2O (in fridge – molecular grade ONLY)

Column 3: EtOH (80% - ENSURE FRESH – on shelf)**

Column 4: EtOH (80% - ENSURE FRESH – on shelf)

Column 5: 0.1 x TE (In fridge; Can be made up from 1.0 x TE if needed)

Column 6: Space for 0.2uL PCR tubes

Column 7: Ampure or NEB next beads (room temperature, mix well before use)

**If running libraries on consecutive days Ethanol should last a few days in botlle, replace EtOH of Reservoir.

5. "Check" that everything is OK and then start the program. Use the following settings:
   i. liquid levels / tube levels – for template plate and 1.5 Eppendorf tubes set all to minimum amount. For adapter plate set volume as estimated. A newly made plate has 50uL of adapter, underestimate so it does not suck air. Reservoirs will have 'detect levels' on, so machine will estimate volumes.
   ii. C4 Labware (y-adaptor plate) – "ignore" (the robot does not recognize this plate, but it is OK to ignore)

6. Once the robot has inspected the setup (last thing it checks is the position of accessories / pipettes), **"pause"** "**HybCap_to96_samples ER and ligation NEBnext"** This will make the thermoplate start and cool to 4°C.

7. Switch the "large metal tube holders" thermoblock in position 1 of the ResRack with a cold one in the fridge.

This is because the robot won't start cooling the pad until it has done the initial check and all the components need to be in place for it to do this. Therefore, we use one thermoblock to ensure that the check can be passed, and then, while the program is paused and the plate is cooling, we switch out the potentially room-temp thermoblock for one that has been kept in the fridge and is definitely cold. This ensures the reagents are always at the right temperature

8. PREPARE **END PREP** - in a 1.5 uL Eppendorf tube **on ice**, mix the **End-Prep Enzyme Mix** reagents (2 vials) according to Table 1., **being sure to use the vortex and centrifuge**. THE **LIGATION MIX** (Stable for 8 hrs) CAN ALSO BE PREPARED DURING THIS STAGE (see table 3)

Table 2 End-repair reaction

Cap Colour	Reagent	Quantity 1X	1X Master Mix (1/2 of original NEBNext II Kit)	48X+8 Master Mix
	Fragmented DNA	1-100 ng	25 uL	Robot needs
(green)	NEBNext Ultra II End Prep Reaction Buffer	7.0ul	3.5 uL	196 *
(green)	NEBNext Ultra II End Prep Enzyme Mix	3.0 ul	1.5 uL	84*
			30 uL	**Dispense 5 ul**

*Added to 1.5mL Eppendorf tube

Caution: The End-repair buffer is **very** viscous. Care should be taken to ensure adequate mixing of the reaction.

9. Prepare **END PREP** reaction **on ice** using the volumes shown above in a single 1.5mL tube and **mix by pipetting up and down**. Spin and place the tube of prepared **End Prep** in position 1A (Lane 1) of the ResRack in thermoblock. Make sure lid is not in the way and point towards you.
10. Resume program **HybCap_to96_samples ER and ligation NEBnext**
11. Once the program has completed the End Prep stage (~45 min), remove the DNA plate from the Robot and cover with bioseal (clear plastic adhesive, Biorad brand).
12. Centrifuge the plate and place in the PCR machine next to robot. Ensure the lid is closed and tightened using the blue rotor.
13. Select "RUN" and then "NNEXT_ER" (incubate 30 min at 20 °C then 30 min at 65 °C)
14. Select "YES" for heated lid and set to 75°C (if possible).
15. Once the incubations have completed (60 min), open lid and remove the bioseal from the plate *ENSURE YOU PULL ACROSS NOT UP!* while the plate is still warm.
16. Place the plate back to the previous position in the robot at 4°C i.e. on thermoblock.

Up until this point you have just repaired the DNA and added an A-tail to the end, next you will ligate the Y-adapters onto the ends of the DNA as shown below:

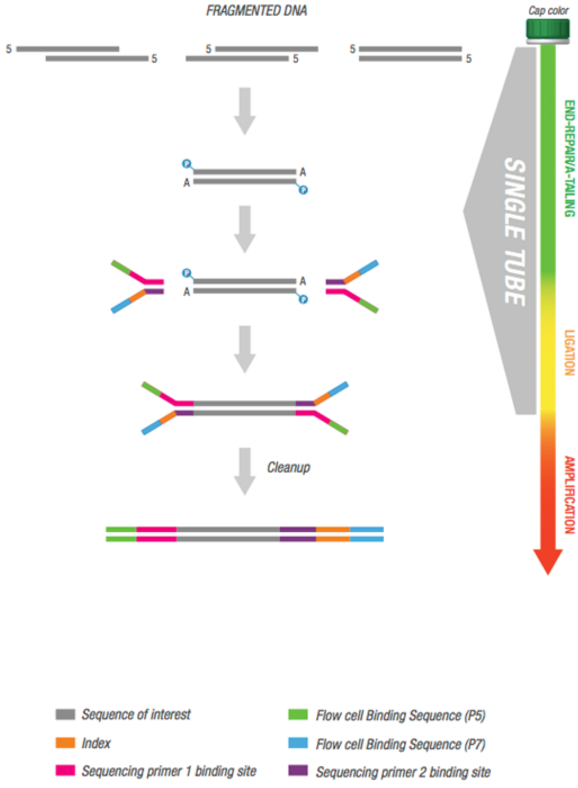

Fig. 1 Workflow for JetSeq™ Flex DNA Library Preparation Kit

Figure 4 This is workflow for Bioline JetSeq, but principle is identical for NEBnextII

Adapter Ligation Set-Up

1. PREPARE **LIGATION MIX** - in a 1.5 uL Eppendorf tube on ice (if not done so already), mix the **Ligation Mix** reagents (2 vials) according to Table 3, **being sure to use the vortex and centrifuge**. **Ligation Mix** can be prepared up to 8 hrs in advance. *note adapters are added by the robot*

Table 3 Adapter Ligation Reaction Mix

Cap Colour	Reagent	1X Master Mix. (1/2 of original NEBNext II Kit)	48X+3 Master Mix
	End-repair reaction from line 10	30uL	X Robot needs 1002ul
Red	NEBNext Ultra II Ligation Master Mix*	15ul	840
Red	NEBNext Ligation Enhancer	0.5ul	28
	Adapters (concentration as required, see table 2)	2ul	Each sample with unique adapter
	Total	47.5 ul	**Dispense 15.5ul**

*Ligation buffer, adapter, ligase and water can be premixed on ice and added in a single pipetting step

2. Place the tube of prepared **Ligation Mix** in position **1B** (Lane 1) of the ResRack in thermoblock
3. Resume "**HybCap_to96_samples ER and ligation NEBnext**"
4. Once the program has completed the Ligation stage (~60 min), remove the DNA plate from the Robot and cover with bioseal (clear plastic adhesive, Biorad brand).
5. Centrifuge the plate and place in the PCR machine. Ensure the lid is closed and tightened using the blue rotor.
6. Select "RUN" and then "NEBN_LIG". (Incubate for 20°C for 20 min)
7. Select "NO" for heated lid.
8. Once the incubation has completed (~20 min), open and remove the bioseal while still in the holder *ENSURE YOU PULL ACROSS NOT UP!*.
9. Transfer the plate to the new position in the robot (see below**)

You then clean up the DNA to remove un-ligated adapters, small sized DNA and adapter-dimers from the library

CLEAN-UP + AMPLIFICATION – "Post-Ligation Clean-Up" + "Library Amplification" (~2.5 HRS WITHOUT GELS)

10. Set up the deck, ensuring all tips have been refilled (Check deck layout on program). Set up a new plate for the Final DNA Sample (LowBind Eppendorf Twin Tec plate) (ensure it is labelled correctly).

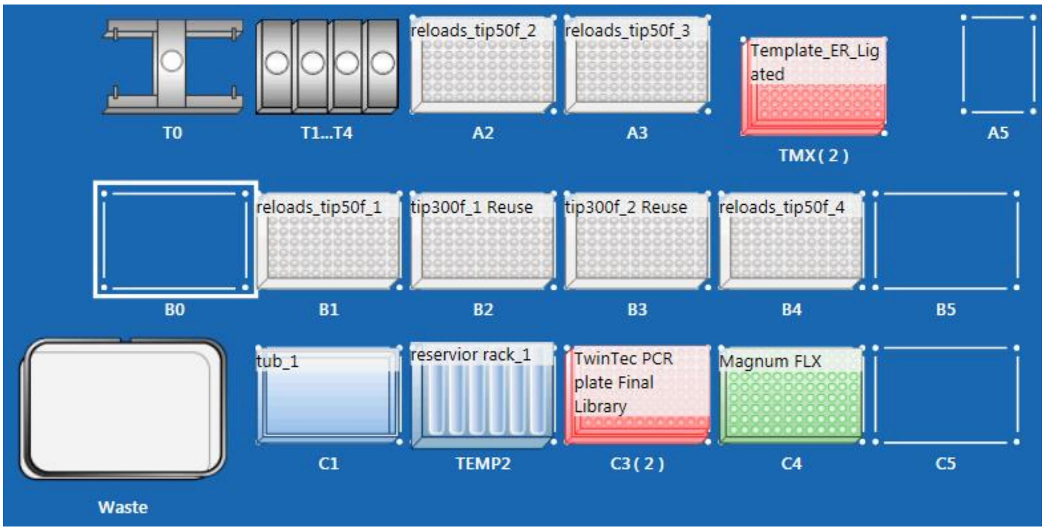

FRONT OF ROBOT

11. Ensure Ampure mix (or NEB Next beads) is at room temperature and has been homogenised by carefully mixing the contents of Lane 7 of the ResRack using the large pipette. (rest of ResRack set up required for this was done earlier). Both Ethanol reservoirs need to be quite full, otherwise program will abort.
12. Load protocol "**HybCap_to96_Post_Lig_cleanup_1x_PCRsetup NEBNext**"

You then need to amplify the library

13. Prepare the Primer Mix by diluting the PreCapture_long primers in nuclease-free water to the final concentration of 10 µM each (NEW this was 2.5uM). Store at -20 °C and thaw on ice before use.- if this was already done skip to next step.
14. PREPARE **MASTER MIX**- in **TWO** 1.5 uL Eppendorf tube on ice, mix the **Master Mix** reagents according to Table 3, being sure to use the vortex and centrifuge.

Table 3: Library amplification reaction

Cap colour	**Reagent**	**Volumes MM 1X**	**X Master Mix (per tube)**
	Purified adapter-ligated library from step 35 (30uL)	16.5uL library	96X samples (+4)
	**NEB Next Ultra ii Q5MM**	17.5 uL	875ul
	Primer Mix **(10 µM each PreCap long primers)**	1 uL	50ul
Total		35 uL	

15. Place the tubes of prepared **Master Mix** in positions 1C & 1D (Lane 1) of the ResRack in thermoblock
16. Begin the next stage of the process in EpMotion.
17. "Check" that everything is OK and then start the program. Use the following settings:
    i. liquid level – set all to minimum amount
    ii. **Master Mix** wells – set to minimum volume.
18. Once the program has completed the clean-up stage and added master mix? (~90 min), remove the Final DNA plate from the Robot and cover with bioseal (clear plastic adhesive, Biorad).
19. Centrifuge the Final DNA plate.
20. Take the plate to the post PCR lab and place in the PCR machine. Ensure the lid is closed.
21. Select "Korjent" as the user, then select "HybCap" and then "NEBNext II" and start the PCR

The conditions of the PCR are as follows

Temperature	Time	Cycles
**98 °C**	30 sec	1
**98 °C**	10 sec	
**65 °C**	75 sec	17 Cycles
**65 °C**	5 min	1
**4 °C**	Hold	

22. Clean up the robot AND SWITCH OFF (or set up for the next run)
    If robot is not switched off thermoblock will stay cold (cooling does not turn off) and deck will get wet.
23. Once the PCR has completed (~45 min) -> IF ENDING PROCESS HERE, store plate in minifridge (MAKE SURE IT IS LABELLED!)

You now have an amplified and purified DNA library that has adapter barcodes on the 5' and 3' ends so contamination is no longer a large issue as anything without a barcode can be identified as contamination. As this is a PCR product the next steps have to be done in the post PCR lab

<u>Observing whether library worked and scoring</u>

1. Run a Gel (large one) on your PCR plate
2. 1.8g agarose + 120mL 1xTBE
3. Into microwave for 1min
4. Cool down agarose by running flask under cold tap. If not, plastic will crack!!
   Agarose is at right temperature if you can just hold it (60°C)
5. Pour into gel set up with combs
6. While this is setting use PCR plates by the sink to set up:
   2uL water
   1uL Midori direct loading dye (6X)
   3uL DNA
7. Once gel has set remove combs

8. Pipette 1uL ladder (tube 100bp ladder with water and Midori green direct) to the first well then add all DNA (6uL) to subsequent wells adding 1uL ladder again to the end well (because it is a large gel ladder should be either side). Don't need to change tip every time but need to rinse between loading.
9. Take photograph of gel
10. Score the samples from 1-4: 1 = best/most DNA 4= worst/leastDNA

The scoring is done relative to the samples not to the ladder and is used next to pool the samples correctly

Pooling samples

1. Pool according to the table below using the scoring from previous section i.e. 1 = 7.5uL, 2 = 15ul, 3 = 22uL and 4 = 30uL (1 = 25%, 2 = 50%, 3 = 75%, 4 = 100%). Pool into 1.5mL low bind Eppendorf tubes. If dealing with difficult DNA or ancient or metabarcoding, pool in sets of 8 samples.

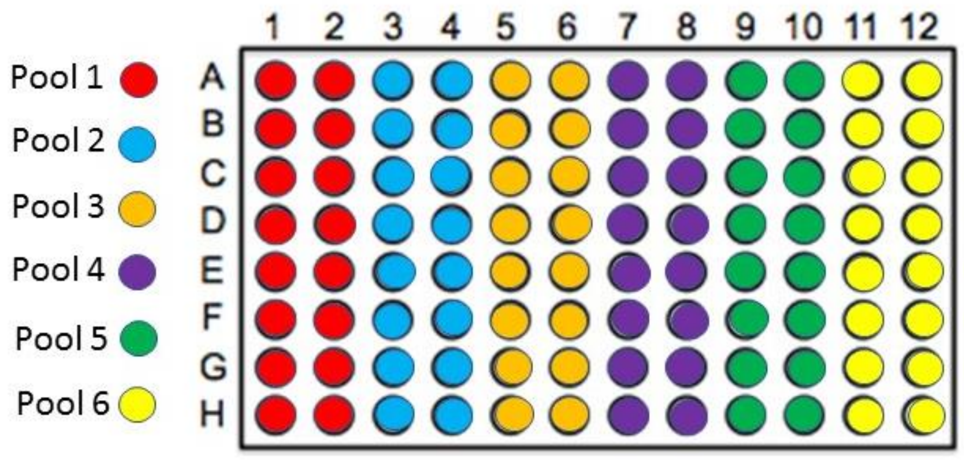

Figure 5 Post Libray Prep pooling scheme. 16 samples per pool 6 pools per plate.

Ampure clean up

This step removes any primer dimers or unwanted PCR products, y adding 0.8x ampure we are removing anything below 300bp as these are too short to sequence anyway

1. Do an Ampure clean up on Pool 1-6
2. Measure the volume in each pool using the pipette technique (suck up liquid then wind down pipette until liquid is at the bottom then read the volume)
3. Add 0.8x the volume of Ampure to each tube, Pipette mix gently 5-10 times.
   The DNA binds to the magnetic beads
4. Incubate at RT 10 min, flash spin and place on magnetic stand and stand for 5-10min.
5. Pipette off supernatant to waste.
   Removing anything not bound to the beads (unwanted products)
6. Wash beads with 500ul of fresh 70% EtOH/ethanol (Prepare fresh!). Stand for 1 min on magnetic bead stand and pipette off supernatant to waste.

This is washing off any unwanted products i.e. purifying your DNA that is still bound to the magnetic beads

7. Repeat step 6.
8. Remove all residual traces of EtOH.
   Do not over-dry the beads as this will decrease yield. The bead pellet is dry when the appearance of the surface changes from shiny to matt.
9. Leave beads to air dry for 5 min.
10. Add 35ul 0.1x TE buffer pipette mix to resuspend beads
    This elutes the DNA from the beads so do not want to get rid of supernatant here!!
11. Incubate at RT 5min
12. Place tubes on magnet for 5 min
13. Carefully pipette supernatant to clean labelled 1.5mL Eppendorf tube - get as much of the buffer as possible, *avoid transferring beads! *

Short version of protocol:

List of samples for this run:

Run code	Well	Sample code	Region	Population	Conc. Ng/ull	Final conc for Fragmentation (ng/ul)
Podocarp03						
Podo_193	A01	Podo_193_Acmopyle_sahniana_Mount_Lofty_Botanic_Park_Living_Collection_ASA_51	0	Mount Lofty Botanic Park Living Collection	0.00	0.00
Podo_194	B01	Podo_194_Acmopyle_pancheri_Mount_Lofty_Botanic_Park_Living_Collection_APA_47	0	Mount Lofty Botanic Park Living Collection	0.00	0.00
Podo_195	C01	Podo_195_Athrotaxis_laxifolia_Mount_Lofty_Botanic_Park_Living_Collection_AL_58	0	Mount Lofty Botanic Park Living Collection	0.00	0.00
Podo_196	D01	Podo_196_Athrotaxis_selaginoides_Mount_Lofty_Botanic_Park_Living_Collection_AS_57	0	Mount Lofty Botanic Park Living Collection	0.00	0.00
Podo_197	E01	Podo_197_Dacrycarpus_imbricatus_Mount_Lofty_Botanic_Park_Living_Collection_DIM_50	0	Mount Lofty Botanic Park Living Collection	0.00	0.00
Podo_198	F01	Podo_198_Dacrydium_beccarii_Mount_Lofty_Botanic_Park_Living_Collection_DB_53	0	Mount Lofty Botanic Park Living Collection	0.00	0.00
Podo_199	G01	Podo_199_Falcatifolium_taxoides_Sp1_large_leaves_Mount_Lofty_Botanic_Park_Living_Collection_FT_S1	0	Mount Lofty Botanic Park Living Collection	0.00	0.00
Podo_200	H01	Podo_200_Falcatifolium_taxoides_Sp2_small_leaves_Mount_Lofty_Botanic_Park_Living_Collection_FT_S2	0	Mount Lofty Botanic Park Living Collection	0.00	0.00
Podo_201	A02	Podo_201_Retrophyllum_comptonii_Mount_Lofty_Botanic_Park_Living_Collection_RC_54	0	Mount Lofty Botanic Park Living Collection	0.00	0.00
Podo_202	B02	Podo_202_Retrophyllum_rospigliosii_Mount_Lofty_Botanic_Park_Living_Collection_RR_55	0	Mount Lofty Botanic Park Living Collection	0.00	0.00
Podo_203	C02	Podo_203_Abies_holophylla_Mount_Lofty_Botanic_Park_Living_Collection_AH_26	0	Mount Lofty Botanic Park Living Collection	0.00	0.00
Podo_204	D02	Podo_204_Abies_procera_Mount_Lofty_Botanic_Park_Living_Collection_AP_25	0	Mount Lofty Botanic Park Living Collection	0.00	0.00
Podo_205	E02	Podo_205_Agathis_australis_Mount_Lofty_Botanic_Park_Living_Collection_AG_1	0	Mount Lofty Botanic Park Living Collection	0.00	0.00
Podo_206	F02	Podo_206_Agathis_microstachya_Mount_Lofty_Botanic_Park_Living_Collection_AME1	0	Mount Lofty Botanic Park Living Collection	0.00	0.00
Podo_207	G02	Podo_207_Araucaria_araucana_Mount_Lofty_Botanic_Park_Living_Collection_AAR_4	0	Mount Lofty Botanic Park Living Collection	0.00	0.00
Podo_208	H02	Podo_208_Callitris_macleayana_Mount_Lofty_Botanic_Park_Living_Collection_CM_9	0	Mount Lofty Botanic Park Living Collection	0.00	0.00
Podo_209	A03	Podo_209_Cedrus_brevifolia_Mount_Lofty_Botanic_Park_Living_Collection_CB_35	0	Mount Lofty Botanic Park Living Collection	0.00	0.00
Podo_210	B03	Podo_210_Cedrus_deodara_Mount_Lofty_Botanic_Park_Living_Collection_CDE_34	0	Mount Lofty Botanic Park Living Collection	0.00	0.00
Podo_211	C03	Podo_211_Cephalotaxus_fortunei_Mount_Lofty_Botanic_Park_Living_Collection_CF_38	0	Mount Lofty Botanic Park Living Collection	0.00	0.00
Podo_212	D03	Podo_212_Cephalotaxus_harringtonia_Mount_Lofty_Botanic_Park_Living_Collection_CHA_37	0	Mount Lofty Botanic Park Living Collection	0.00	0.00
Podo_213	E03	Podo_213_Chamaecyparis_pisifera_Mount_Lofty_Botanic_Park_Living_Collection_CP_16	0	Mount Lofty Botanic Park Living Collection	0.00	0.00
Podo_214	F03	Podo_214_Chamaecyparis_thyoides_Mount_Lofty_Botanic_Park_Living_Collection_CT_15	0	Mount Lofty Botanic Park Living Collection	0.00	0.00
Podo_215	G03	Podo_215_Cryptomeria_japonica_Mount_Lofty_Botanic_Park_Living_Collection_CJ_24	0	Mount Lofty Botanic Park Living Collection	0.00	0.00
Podo_216	H03	Podo_216_Cycas_revoluta_Mount_Lofty_Botanic_Park_Living_Collection_CR_44	0	Mount Lofty Botanic Park Living Collection	0.00	0.00
Podo_217	A04	Podo_217_Diselma_archeri_Mount_Lofty_Botanic_Park_Living_Collection_DA_12	0	Mount Lofty Botanic Park Living Collection	0.00	0.00
Podo_218	B04	Podo_218_Fitzroya_cupressoides_Mount_Lofty_Botanic_Park_Living_Collection_FC_13	0	Mount Lofty Botanic Park Living Collection	0.00	0.00
Podo_219	C04	Podo_219_Ginkgo_biloba_Mount_Lofty_Botanic_Park_Living_Collection_GG_1	0	Mount Lofty Botanic Park Living Collection	0.00	0.00
Podo_220	D04	Podo_220_Juniperus_cummunis_Mount_Lofty_Botanic_Park_Living_Collection_JU_21	0	Mount Lofty Botanic Park Living Collection	0.00	0.00
Podo_221	E04	Podo_221_Juniperus_oxycedrus_Mount_Lofty_Botanic_Park_Living_Collection_JO_20	0	Mount Lofty Botanic Park Living Collection	0.00	0.00
Podo_222	F04	Podo_222_Keteleeria_evelyniana_Mount_Lofty_Botanic_Park_Living_Collection_ke_1	0	Mount Lofty Botanic Park Living Collection	0.00	0.00
Podo_223	G04	Podo_223_Picea_glauca_Mount_Lofty_Botanic_Park_Living_Collection_PG_28	0	Mount Lofty Botanic Park Living Collection	0.00	0.00
Podo_224	H04	Podo_224_Picea_smithiana_Mount_Lofty_Botanic_Park_Living_Collection_PS_27	0	Mount Lofty Botanic Park Living Collection	0.00	0.00
Podo_225	A05	Podo_225_Pinus_brutia_Mount_Lofty_Botanic_Park_Living_Collection_PB_33	0	Mount Lofty Botanic Park Living Collection	0.00	0.00
Podo_226	B05	Podo_226_Pinus_contorta_Mount_Lofty_Botanic_Park_Living_Collection_PC_32	0	Mount Lofty Botanic Park Living Collection	0.00	0.00
Podo_227	C05	Podo_227_Pseudotsuga_menziesii_Mount_Lofty_Botanic_Park_Living_Collection_PM_29	0	Mount Lofty Botanic Park Living Collection	0.00	0.00
Podo_228	D05	Podo_228_Pseudotsuga_sinensis_Mount_Lofty_Botanic_Park_Living_Collection_PS_30	0	Mount Lofty Botanic Park Living Collection	0.00	0.00
Podo_229	E05	Podo_229_Sciadopitys_verticillata_Mount_Lofty_Botanic_Park_Living_Collection_SV_36	0	Mount Lofty Botanic Park Living Collection	0.00	0.00
Podo_230	F05	Podo_230_Sequoia_sempervirens_Mount_Lofty_Botanic_Park_Living_Collection_SS_22	0	Mount Lofty Botanic Park Living Collection	0.00	0.00
Podo_231	G05	Podo_231_Sequoiadendron_giganteum_Mount_Lofty_Botanic_Park_Living_Collection_SG_23	0	Mount Lofty Botanic Park Living Collection	0.00	0.00
Podo_232	H05	Podo_232_Stangeria_eriopus_Mount_Lofty_Botanic_Park_Living_Collection_SE_46	0	Mount Lofty Botanic Park Living Collection	0.00	0.00
Podo_233	A06	Podo_233_Sundacarpus_amarus_Mount_Lofty_Botanic_Park_Living_Collection_SA_1	0	Mount Lofty Botanic Park Living Collection	0.00	0.00
Podo_234	B06	Podo_234_Taxus_baccata_Mount_Lofty_Botanic_Park_Living_Collection_TB_39	0	Mount Lofty Botanic Park Living Collection	0.00	0.00
Podo_235	C06	Podo_235_Thuja_occidentalis_Mount_Lofty_Botanic_Park_Living_Collection_TO_14	0	Mount Lofty Botanic Park Living Collection	0.00	0.00
Podo_236	D06	Podo_236_Torreya_nucifera_Mount_Lofty_Botanic_Park_Living_Collection_TN_1	0	Mount Lofty Botanic Park Living Collection	0.00	0.00
Podo_237	E06	Podo_237_Tsuga_canadensis_Mount_Lofty_Botanic_Park_Living_Collection_TC_31	0	Mount Lofty Botanic Park Living Collection	0.00	0.00
Podo_238	F06	Podo_238_Widdringtonia_schwarzii_Mount_Lofty_Botanic_Park_Living_Collection_WS_10	0	Mount Lofty Botanic Park Living Collection	0.00	0.00
Podo_239	G06	Podo_239_Widdringtonia_wallichii_Mount_Lofty_Botanic_Park_Living_Collection_WW_11	0	Mount Lofty Botanic Park Living Collection	0.00	0.00
Podo_240	H06	Podo_240_Wollemia_nobilis_Mount_Lofty_Botanic_Park_Living_Collection_WN_5	0	Mount Lofty Botanic Park Living Collection	0.00	0.00

Table 4 End-repair reaction

Cap Colour	*Reagent*	*Quantity 1X*	*1X Master Mix (1/2 of original NEBNext II Kit)*	*48X+8 Master Mix*
	Fragmented DNA	1-100 ng	25 uL	Robot needs
	NEBNext Ultra II End Prep Reaction Buffer	7.0ul	3.5 uL	196 *
	NEBNext Ultra II End Prep Enzyme Mix	3.0 ul	1.5 uL	84*
			30 uL	**Dispense 5 ul**

*Added to 1.5mL Eppendorf tube

Table 5 Adapter Ligation Reaction Mix

Cap Colour	*Reagent*	**1X Master Mix.** *(1/2 of original NEBNext II Kit)*	**48X+3 Master Mix**
	End-repair reaction from line 10	30uL	X<br>Robot needs 1002ul
	NEBNext Ultra II Ligation Master Mix*	15ul	840
	NEBNext Ligation Enhancer	0.5ul	28
	Adapters (concentration as required, see table 2)	2ul	Each sample with unique adapter
	Total	47.5 ul	**Dispense 15.5ul**

*Ligation buffer, adapter, ligase and water can be premixed on ice and added in a single pipetting step

Cap colour	**Reagent**	**Volumes MM 1X**	**X Master Mix (per tube)**
	Purified adapter-ligated library from step 35 (30uL)	16.5uL library	48X samples (+4)
	**NEB Next Ultra ii Q5MM**	17.5 uL	875ul
	Primer Mix **(10 µM each PreCap long primers)**	1 uL	50ul
Total		35 uL	

Temperature	Time	Cycles
**98 °C**	30 sec	1
**98 °C**	10 sec	
**65 °C**	20 sec	19 Cycles
**72 °C**	20 sec	
**72 °C**	2 min	1
**4 °C**	Hold	

Gel Images from this run

Figure 6 Put 3ul library on 1.5 agarose gel

Barcode and Index details

Sample	Well	pool	ul in pool	Barcode	internal barcode	Index name i7	Index i7	Index name i5	Index i5
**Podocarp03**									
Podo_193_Acmopyle_sahniana_Mount_Lofty_Botanic_Park_Living_Collection_ASA_51	A01	76-1		AACAACCG	trcY_Tag001	P7_primer_Idx004	GCTACAAC	P5_primer_Idx009	CCAACACT
Podo_194_Acmopyle_pancheri_Mount_Lofty_Botanic_Park_Living_Collection_APA_47	B01	76-1		CACGTCTA	trcY_Tag002	P7_primer_Idx004	GCTACAAC	P5_primer_Idx009	CCAACACT
Podo_195_Athrotaxis_laxifolia_Mount_Lofty_Botanic_Park_Living_Collection_AL_58	C01	76-1		TTCCTCCT	trcY_Tag003	P7_primer_Idx004	GCTACAAC	P5_primer_Idx009	CCAACACT
Podo_196_Athrotaxis_selaginoides_Mount_Lofty_Botanic_Park_Living_Collection_AS_57	D01	76-1		GCTACAAC	trcY_Tag004	P7_primer_Idx004	GCTACAAC	P5_primer_Idx009	CCAACACT
Podo_197_Dacrycarpus_imbricatus_Mount_Lofty_Botanic_Park_Living_Collection_DIM_50	E01	76-1		CGACACTT	trcY_Tag005	P7_primer_Idx004	GCTACAAC	P5_primer_Idx009	CCAACACT
Podo_198_Dacrydium_beccarii_Mount_Lofty_Botanic_Park_Living_Collection_DB_53	F01	76-1		GATCTTGC	trcY_Tag006	P7_primer_Idx004	GCTACAAC	P5_primer_Idx009	CCAACACT
Podo_199_Falcatifolium_taxoides_Sp1_large_leaves_Mount_Lofty_Botanic_Park_Living_Collection_FT_S1	G01	76-1		TGCTTGCT	trcY_Tag007	P7_primer_Idx004	GCTACAAC	P5_primer_Idx009	CCAACACT
Podo_200_Falcatifolium_taxoides_Sp2_small_leaves_Mount_Lofty_Botanic_Park_Living_Collection_FT_S2	H01	76-1		CCAACACT	trcY_Tag008	P7_primer_Idx004	GCTACAAC	P5_primer_Idx009	CCAACACT
Podo_201_Retrophyllum_comptonii_Mount_Lofty_Botanic_Park_Living_Collection_RC_54	A02	76-1		CTAGCTCA	trcY_Tag009	P7_primer_Idx004	GCTACAAC	P5_primer_Idx009	CCAACACT
Podo_202_Retrophyllum_rospigliosii_Mount_Lofty_Botanic_Park_Living_Collection_RR_55	B02	76-1		ATCATGCG	trcY_Tag010	P7_primer_Idx004	GCTACAAC	P5_primer_Idx009	CCAACACT
Podo_203_Abies_holophylla_Mount_Lofty_Botanic_Park_Living_Collection_AH_26	C02	76-1		CAGCTAGA	trcY_Tag011	P7_primer_Idx004	GCTACAAC	P5_primer_Idx009	CCAACACT
Podo_204_Abies_procera_Mount_Lofty_Botanic_Park_Living_Collection_AP_25	D02	76-1		TTGCGAGA	trcY_Tag012	P7_primer_Idx004	GCTACAAC	P5_primer_Idx009	CCAACACT
Podo_205_Agathis_australis_Mount_Lofty_Botanic_Park_Living_Collection_AG_1	E02	76-1		GGCGAATA	trcY_Tag013	P7_primer_Idx004	GCTACAAC	P5_primer_Idx009	CCAACACT
Podo_206_Agathis_microstachya_Mount_Lofty_Botanic_Park_Living_Collection_AME1	F02	76-1		TACGACGT	trcY_Tag014	P7_primer_Idx004	GCTACAAC	P5_primer_Idx009	CCAACACT
Podo_207_Araucaria_araucana_Mount_Lofty_Botanic_Park_Living_Collection_AAR_4	G02	76-1		CCAACTTC	trcY_Tag015	P7_primer_Idx004	GCTACAAC	P5_primer_Idx009	CCAACACT
Podo_208_Callitris_macleayana_Mount_Lofty_Botanic_Park_Living_Collection_CM_9	H02	76-1		AGACATGC	trcY_Tag016	P7_primer_Idx004	GCTACAAC	P5_primer_Idx009	CCAACACT
Podo_209_Cedrus_brevifolia_Mount_Lofty_Botanic_Park_Living_Collection_CB_35	A03	76-2		CCTATTGG	trcY_Tag017	P7_primer_Idx004	GCTACAAC	P5_primer_Idx009	CCAACACT
Podo_210_Cedrus_deodara_Mount_Lofty_Botanic_Park_Living_Collection_CDE_34	B03	76-2		TCTAGGAG	trcY_Tag018	P7_primer_Idx004	GCTACAAC	P5_primer_Idx009	CCAACACT
Podo_211_Cephalotaxus_fortunei_Mount_Lofty_Botanic_Park_Living_Collection_CF_38	C03	76-2		GATCTCAG	trcY_Tag019	P7_primer_Idx004	GCTACAAC	P5_primer_Idx009	CCAACACT
Podo_212_Cephalotaxus_harringtonia_Mount_Lofty_Botanic_Park_Living_Collection_CHA_37	D03	76-2		AACTTGCC	trcY_Tag020	P7_primer_Idx004	GCTACAAC	P5_primer_Idx009	CCAACACT
Podo_213_Chamaecyparis_pisifera_Mount_Lofty_Botanic_Park_Living_Collection_CP_16	E03	76-2		ACACGAGA	trcY_Tag021	P7_primer_Idx004	GCTACAAC	P5_primer_Idx009	CCAACACT
Podo_214_Chamaecyparis_thyoides_Mount_Lofty_Botanic_Park_Living_Collection_CT_15	F03	76-2		CATCCAAG	trcY_Tag022	P7_primer_Idx004	GCTACAAC	P5_primer_Idx009	CCAACACT
Podo_215_Cryptomeria_japonica_Mount_Lofty_Botanic_Park_Living_Collection_CJ_24	G03	76-2		CAAGGTAC	trcY_Tag023	P7_primer_Idx004	GCTACAAC	P5_primer_Idx009	CCAACACT
Podo_216_Cycas_revoluta_Mount_Lofty_Botanic_Park_Living_Collection_CR_44	H03	76-2		GTCCTAAG	trcY_Tag024	P7_primer_Idx004	GCTACAAC	P5_primer_Idx009	CCAACACT
Podo_217_Diselma_archeri_Mount_Lofty_Botanic_Park_Living_Collection_DA_12	A04	76-2		CCTCATCT	trcY_Tag025	P7_primer_Idx004	GCTACAAC	P5_primer_Idx009	CCAACACT
Podo_218_Fitzroya_cupressoides_Mount_Lofty_Botanic_Park_Living_Collection_FC_13	B04	76-2		AATCCAGC	trcY_Tag026	P7_primer_Idx004	GCTACAAC	P5_primer_Idx009	CCAACACT
Podo_219_Ginkgo_biloba_Mount_Lofty_Botanic_Park_Living_Collection_GG_1	C04	76-2		ACAGTTCG	trcY_Tag027	P7_primer_Idx004	GCTACAAC	P5_primer_Idx009	CCAACACT
Podo_220_Juniperus_cummunis_Mount_Lofty_Botanic_Park_Living_Collection_JU_21	D04	76-2		CAACTCCA	trcY_Tag028	P7_primer_Idx004	GCTACAAC	P5_primer_Idx009	CCAACACT
Podo_221_Juniperus_oxycedrus_Mount_Lofty_Botanic_Park_Living_Collection_JO_20	E04	76-2		CAATGCGA	trcY_Tag029	P7_primer_Idx004	GCTACAAC	P5_primer_Idx009	CCAACACT
Podo_222_Keteleeria_evelyniana_Mount_Lofty_Botanic_Park_Living_Collection_ke_1	F04	76-2		GATGGAGT	trcY_Tag030	P7_primer_Idx004	GCTACAAC	P5_primer_Idx009	CCAACACT
Podo_223_Picea_glauca_Mount_Lofty_Botanic_Park_Living_Collection_PG_28	G04	76-2		TTCGAAGC	trcY_Tag031	P7_primer_Idx004	GCTACAAC	P5_primer_Idx009	CCAACACT
Podo_224_Picea_smithiana_Mount_Lofty_Botanic_Park_Living_Collection_PS_27	H04	76-2		CCGAAGAT	trcY_Tag032	P7_primer_Idx004	GCTACAAC	P5_primer_Idx009	CCAACACT
Podo_225_Pinus_brutia_Mount_Lofty_Botanic_Park_Living_Collection_PB_33	A05	76-3		ACTGCTTG	trcY_Tag033	P7_primer_Idx004	GCTACAAC	P5_primer_Idx009	CCAACACT
Podo_226_Pinus_contorta_Mount_Lofty_Botanic_Park_Living_Collection_PC_32	B05	76-3		AGGTTCCT	trcY_Tag034	P7_primer_Idx004	GCTACAAC	P5_primer_Idx009	CCAACACT
Podo_227_Pseudotsuga_menziesii_Mount_Lofty_Botanic_Park_Living_Collection_PM_29	C05	76-3		ACGCAGTA	trcY_Tag035	P7_primer_Idx004	GCTACAAC	P5_primer_Idx009	CCAACACT
Podo_228_Pseudotsuga_sinensis_Mount_Lofty_Botanic_Park_Living_Collection_PS_30	D05	76-3		GGACTACT	trcY_Tag036	P7_primer_Idx004	GCTACAAC	P5_primer_Idx009	CCAACACT
Podo_229_Sciadopitys_verticillata_Mount_Lofty_Botanic_Park_Living_Collection_SV_36	E05	76-3		GGCATTCT	trcY_Tag037	P7_primer_Idx004	GCTACAAC	P5_primer_Idx009	CCAACACT
Podo_230_Sequoia_sempervirens_Mount_Lofty_Botanic_Park_Living_Collection_SS_22	F05	76-3		CACAGACT	trcY_Tag038	P7_primer_Idx004	GCTACAAC	P5_primer_Idx009	CCAACACT
Podo_231_Sequoiadendron_giganteum_Mount_Lofty_Botanic_Park_Living_Collection_SG_23	G05	76-3		CGATTGGA	trcY_Tag039	P7_primer_Idx004	GCTACAAC	P5_primer_Idx009	CCAACACT
Podo_232_Stangeria_eriopus_Mount_Lofty_Botanic_Park_Living_Collection_SE_46	H05	76-3		GACTTGTG	trcY_Tag040	P7_primer_Idx004	GCTACAAC	P5_primer_Idx009	CCAACACT
Podo_233_Sundacarpus_amarus_Mount_Lofty_Botanic_Park_Living_Collection_SA_1	A06	76-3		GAGGCATT	trcY_Tag041	P7_primer_Idx004	GCTACAAC	P5_primer_Idx009	CCAACACT
Podo_234_Taxus_baccata_Mount_Lofty_Botanic_Park_Living_Collection_TB_39	B06	76-3		GCGTTAGA	trcY_Tag042	P7_primer_Idx004	GCTACAAC	P5_primer_Idx009	CCAACACT
Podo_235_Thuja_occidentalis_Mount_Lofty_Botanic_Park_Living_Collection_TO_14	C06	76-3		ACGGACTT	trcY_Tag043	P7_primer_Idx004	GCTACAAC	P5_primer_Idx009	CCAACACT
Podo_236_Torreya_nucifera_Mount_Lofty_Botanic_Park_Living_Collection_TN_1	D06	76-3		ATTAGCCG	trcY_Tag044	P7_primer_Idx004	GCTACAAC	P5_primer_Idx009	CCAACACT
Podo_237_Tsuga_canadensis_Mount_Lofty_Botanic_Park_Living_Collection_TC_31	E06	76-3		CACAGGAA	trcY_Tag045	P7_primer_Idx004	GCTACAAC	P5_primer_Idx009	CCAACACT
Podo_238_Widdringtonia_schwarzii_Mount_Lofty_Botanic_Park_Living_Collection_WS_10	F06	76-3		AGGAACAC	trcY_Tag046	P7_primer_Idx004	GCTACAAC	P5_primer_Idx009	CCAACACT
Podo_239_Widdringtonia_wallichii_Mount_Lofty_Botanic_Park_Living_Collection_WW_11	G06	76-3		AGCCGTAA	trcY_Tag047	P7_primer_Idx004	GCTACAAC	P5_primer_Idx009	CCAACACT
Podo_240_Wollemia_nobilis_Mount_Lofty_Botanic_Park_Living_Collection_WN_5	H06	76-3		ACTCTCCA	trcY_Tag048	P7_primer_Idx004	GCTACAAC	P5_primer_Idx009	CCAACACT
